# *Fasciola hepatica*: A cause of Obstructive Jaundice in an Elderly Man From Iran

**DOI:** 10.4103/1319-3767.43279

**Published:** 2008-10

**Authors:** Mohsen Moghadami, M. Mardani

**Affiliations:** Department of Internal Medicine, Shiraz Medical School, Shiraz, Iran; 1 Infectious Disease Research Center, Shahid Beheshti University of Medical Science, Tehran, Iran

**Keywords:** *Fasciola*, jaundice, Iran, obstruction

## Abstract

Fascioliasis is a zoonotic infection caused by *Fasciola hepatica*. Humans can become accidental hosts of this parasite by ingesting contaminated drinking water or plants in endemic area. The north of Iran is one of the regions. This disease is rarely seen with jaundice caused by obstruction of the biliary tree. We report a case of human fascioliasis with obstructive jaundice who was diagnosed using endoscopic retrograde cholangiopancreatography (ERCP). This report confirms the diagnostic role of ERCP in patients with obstructive jaundice caused by biliary fascioliasis.

Human fascioliasis is a zoonosis caused by ***Fasciola hepatica*** (***F. hepatica***), a trematode that infests cattle and sheep. Humans are accidental hosts in the life cycle of this parasite. It is reported that 2.5 million people have been infected in 61 countries especially from Bolivia, Peru, Egypt, Iran, Portugal, and France, and that more than 180 million people are at risk.[1]

In the north of Iran, carpological studies showed 7.3 and 25.4% global prevalence in sheep and cattle, respectively, and traditions in herbal condiments for human consumption, methods of animal husbandry, and annual rainfall may explain the higher prevalence in comparison with other geographic regions of Iran.[2]

Infestation with ***F. hepatica*** has a variable clinical presentation. In acute phase, symptoms corresponding migration of larval stage from intestine and in chronic phase symptoms are subclinical, and only intermittent cholangitis may be the prominent sign. We describe a patient who presented with obstructive jaundice that had been caused by ***F. hepatica***.

## CASE REPORT

**Figure 1:**
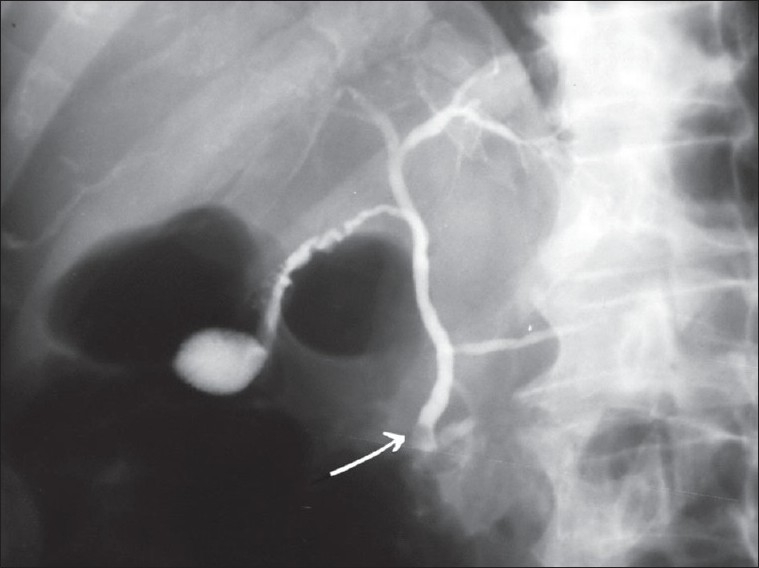
*Fasciola* caused an obstructive mass in common bile duct

A 73-year-old man, farmer from Gorgan, a city in the north of Iran, who came because of fever and icterus since 1 week before presentation. He had no obvious symptoms since 1 week before admission when he developed progressive jaundice and episodic right upper quadrant pain especially after meal but unrelated to position or respiration. He also reported mild fever, anorexia, and generalized body pain during this period. To analyze his problem, abdominal sonography was performed in outpatient setting, which showed an increased liver echogenesity without the dilation of bile ducts and gall stone. Based oh the abovementioned symptoms, his primary care physician made a diagnosis of viral hepatitis, but the patient eventually developed constant high-grade fever and right upper quadrant pain. Therefore, he was referred to our hospital and was admitted. At the time of hospitalization, the temperature was 39.5°C, the pulse was 90 beats/min, and the respiratory rate was 18 breaths/min. The blood pressure was 110/65 mm Hg. On physical examination, the patient did not appear to be in severe pain, and there was mild jaundice. The lungs and heart sounds were normal. The abdomen was flat, and the bowel sounds were present. There was tenderness in the right upper and quadrant, with mild guarding but without rebound tenderness. No mass or hernia was detected. The arms and legs were well perfused. No abnormalities were found on rectal examination. His sonography at admission revealed minimal intrahepatic and mild extrahepatic biliary dilation, while other organs were normal and no stones were observed. Blood tests revealed mild normocytic normochromic anemia, while white blood cell and absolute eosinophil count were normal in the patient. The erythrocyte sedimentation rates were high (90 mm/h) and serum chemical analysis revealed the following values: albumin, 3.4 g/dl; alkaline phosphatase, 170 U/l (normal range, 53–128); alanine aminotransferase, 73 U/l (normal range, 10–35); aspartate aminotransferase, 54 U/l (normal range, 14–50); and total bilirubin, 8.9 mg/dl (normal range 0.3–1.2), with a direct bilirubin level of 5.7 mg/dl (normal,< 0.2). The white-cell count was 11 400/mm3, with 84% neutrophils and 1% eosinophils. The urine was orange and was positive for bile (++), protein (+), and urobilinogen (+). Because of the endemicity of leptospirosis, serologic test (indirect hemagglutination test) was done, which revealed negative result. A urine culture and multiple blood cultures were negative. Assays for the presence of hepatitis B antigen and antibody, hepatitis C antibody, and antimitochondrial antibodies were negative. Chest radiographs showed no abnormalities. Endoscopic retrograde cholangiopancreatography (ERCP) demonstrated extrahepatic biliary dilatation; the common bile ducts (CBDs) were about 12–15 mm in diameter, with a small filling defect in the distal part [Figure 1].

Serologic test for ***F. hepatica*** showed high titer against that (positive titer 1/80), and the detection of ova in stool documented infestation with this liver fluke. After diagnosis, triclabendazole was administered at a dose of 10–12 mg/kg for 1 or 2 days, after which the symptoms disappeared and biochemical values soon returned to normal, and the patient was discharged with good condition. During the follow-up, her jaundice subsided, and ultrasonography revealed an almost normal CBD in our patient.

## DISCUSSION

Although fascioliasis was seen mainly in developing countries, in the last decade, the number of cases in the developed countries has increased, reaching 61 countries worldwide because of the increase in worldwide traveling and immigration. Iran, especially the northern area with temperate climate, is an endemic region for this disease. The fascioliasis situation in humans and livestock of Iranian Mazandaran (North province of Iran) showed 7.3 and 25.4% global prevalence in sheep and cattle, respectively. Studies in slaughterhouses indicate that sheep and cattle may be the main reservoir species.[3] The case reported in this article came from this area.

Humans usually get infection by eating watercress grown in sheep-raising areas. Other freshwater plants may also transmit the infection. Humans are also occasionally infected by drinking unboiled contaminated water containing viable metacercariae[4] or by eating poorly cooked liver of sheep. When eating infected material, infective metacercariae excyst in the duodenum and larvae emerge. The larvae penetrate the wall of the small intestine into the peritoneal cavity, then penetrate the liver capsule, and pass through the liver tissue into the biliary tract.

Typical symptoms that may be associated with fascioliasis can be divided by phases of the disease including the acute or liver phase, the chronic or biliary phase, and ectopic or pharyngeal fascioliasis.[5] The acute (or hepatic) stage is characterized by fever, abdominal pain, headache, pruritis, urticaria, weight loss, and eosinophilia. Transaminase levels are in normal range or are only minimally elevated, and bilirubin levels are typically in normal range. The biliary phase is usually asymptomatic, but it is rarely reported in the medical literature that it can lead to extrahepatic obstruction and cholestasis, as was the case with our patient. In a report published in 2000, only 19 cases were reported to have had CBD obstruction by ***F. hepatica*** during the last 10 years.[6] Again at 2006, Gulsen ***et al.*** reported five cases who were admitted to hospital with complaints of icterus and pain in the right upper quadrant of the abdomen, and ERCP showed the presence of ***F. hepatica*** in the CBD.[7]

Besides the direct obstructive effect of the fluke over bile ducts, secondary fibrosis and stricture formation can be seen during chronic infestation.[8]

The diagnosis can be made by finding characteristic ova in feces, duodenal aspirates, or bile specimens.[9] During the first 3-4 months of acute infection, immunologic techniques play an important role in the diagnosis of fascioliasis. An enzyme-linked immunosorbent assay (ELISA) has a sensitivity of 100% and a specificity of 97.8%.[10] During the early larval stage of infection, eggs are not found in the stool. However, stool can be examined for eggs during the biliary stage of infection. Eggs are nonembryonated, ovoid, and large (130–150 × 60– 90 *μ*m2), with a small operculum. The egg looks nearly identical to that of ***F.*** buski (an intestinal trematode). Because eggs are released sporadically, the number of eggs excreted can vary widely, and it may be necessary to examine multiple concentrated stool specimens.[11] With the abovementioned case reports, we should consider the role of imaging studies such as ERCP in the diagnosis of infestation.

Many drugs have been used to treat fascioliasis with varying success. Unlike infection with other trematodes, fascioliasis responds poorly to praziquantel. A first-line treatment is with a single dose of triclabendazole, a well-tolerated benzimidazole used in daily practice that is highly effective against mature and immature flukes.[12] Triclabendazole can be given as a single oral dose of 10 mg/kg or, in cases of severe infection, in two 10 mg/kg doses given 12 h apart. Triclabendazole should be administered with food to increase its bioavailability.[13]

In conclusion, because of the immigration worldwide, and especially in endemic countries such as Iran, physicians should be aware of this disease and they should keep in mind the differential diagnosis of obstructive jaundice. Also, ERCP is still important in the diagnosis and treatment of the disease and can be used safely.
